# Longitudinal Associations of Modifiable Lifestyle Factors With Positive Depression-Screen Over 2.5-Years in an International Cohort of People Living With Multiple Sclerosis

**DOI:** 10.3389/fpsyt.2018.00526

**Published:** 2018-10-30

**Authors:** Keryn L. Taylor, Steve Simpson, George A. Jelinek, Sandra L. Neate, Alysha M. De Livera, Chelsea R. Brown, Emily O'Kearney, Claudia H. Marck, Tracey J. Weiland

**Affiliations:** ^1^Neuroepidemiology Unit, Melbourne School of Population and Global Health, University of Melbourne, Carlton, VIC, Australia; ^2^Department of Psychiatry and Psychosocial Cancer Care, St Vincent's Hospital Melbourne, Fitzroy, VIC, Australia; ^3^Menzies Institute for Medical Research, University of Tasmania, Hobart, TAS, Australia; ^4^Centre for Health Equity, Melbourne School of Population and Global Health, The University of Melbourne, Carlton, VIC, Australia

**Keywords:** Multiple sclerosis, epidemiology, depression, lifestyle, longitudinal, cohort study (or longitudinal study)

## Abstract

**Background:** Depression is common and has a significant impact on quality of life for many people with multiple sclerosis (MS). A preventive management approach via modification of lifestyle risk factors holds potential benefits. We examined the relationship between modifiable lifestyle factors and depression risk and the change in depression over 2.5 years.

**Methods:** Sample recruited using online platforms. 2,224 (88.9%) at baseline and 1,309 (93.4%) at 2.5 years follow up completed the necessary survey data. Depression risk was measured by the Patient Health Questionnaire-2 (PHQ-2) at baseline and Patient Health Questionniare-9 (PHQ-9) at 2.5-years follow-up. Multivariable regression models assessed the relationships between lifestyle factors and depression risk, adjusted for sex, age, fatigue, disability, antidepressant medication use, and baseline depression score, as appropriate.

**Results:** The prevalence of depression risk at 2.5-years follow-up in this cohort was 14.5% using the PHQ-2 and 21.7% using the PHQ-9. Moderate alcohol intake, being a non-smoker, diet quality, no meat or dairy intake, vitamin D supplementation, omega 3 supplement use, regular exercise, and meditation at baseline were associated with lower frequencies of positive depression-screen 2.5 years later. Moderate alcohol intake was associated with greater likelihood of becoming depression-free and a lower likelihood of becoming depressed at 2.5-years follow-up. Meditating at least once a week was associated with a decreased frequency of losing depression risk, against our expectation. After adjusting for potential confounders, smoking, diet, physical activity, and vitamin D and omega-3 supplementation were not associated with a change in risk for depression.

**Conclusion:** In a large prospective cohort study of people with MS and depression, in line with the emerging treatment paradigm of early intervention, these results suggest a role for some lifestyle factors in depression risk. Further studies should endeavor to explore the impact of positive lifestyle change and improving depression in people living with MS.

## Introduction

Multiple sclerosis (MS) is an autoimmune, demyelinating, inflammatory disease of the central nervous system. Symptoms are diverse and can include sensory, motor and visual deficits, bowel and bladder dysfunction, cognitive impairment and fatigue. Neuropsychiatric symptoms are also common, with an episode of depression occurring for 50% of people with MS during their lifetime (1); the annual prevalence of depression has been estimated as 20% (2), increasing to 25% among people in the 18–45 years old age range (3).

In addition to its negative impacts on overall quality of life, depression is associated with suicide, a significant cause of mortality for people with MS (4). People with MS have both a higher suicide rate (5) and all-cause mortality rate compared to the general population (4), with up to 35% of people with MS reporting suicidal ideation during their lifetime (6). Suicidal ideation is strongly associated with depression and can be present even if depressive symptoms are mild (6). However, a greater severity of depression shows an even stronger association with suicidal thoughts (7). Suicidal ideation is associated with both actual and perceived disability, with depression a mediating factor in this relationship. Depression is therefore a modifiable factor, potentially via both prevention and treatment, to reduce the risk of suicide and improve mortality outcomes for people with MS (6).

The evidence base for treatment of depression for people with MS is limited. Pharmacological and psychological treatment are the mainstay in management: prescription of antidepressant medication for people with MS is widespread and there is a clear need to establish the evidence base behind this practice (8). The most recent Cochrane review of pharmacological treatment reported a trend toward efficacy for two antidepressant medications, but cautioned about the significant methodological problems of the research, high rates of adverse effects, and issues regarding loss to follow-up which may affect generalizability (9). A more recent review of clinical trials recommended antidepressant medication choice be based on the medication side effect profile and tailored for the individual, as the side effects of antidepressant medications can worsen common symptoms of MS, such as fatigue, sexual dysfunction, and bowel/bladder dysfunction (10). Most non-pharmacological research investigating the management of depression in MS has focused on psychological interventions, particularly cognitive behavioral therapy (11). A recent meta-analysis of cognitive behavioral therapy showed a moderate effect on depression in the short term for people with MS (11). Exercise therapy for depression is increasingly common and effective in the general population (12), and has also shown promise for people with MS (13). Mindfulness-based interventions for people with MS have also been shown to improve depression and quality of life at 6 months follow-up (14).

The challenge of treating depression extends to the general population where pharmacological and psychological interventions are also first-line treatments, yet have limited impact, only reducing the burden of depression by 10–30% (15). An emerging paradigm which provides a nexus between prevention, health promotion and clinical treatment of depression, is modification of known lifestyle risk factors for depression (16, 17). In the general population, prospective studies in teenagers and adults showed that modification of lifestyle factors, including diet, exercise, weight and smoking, improved and prevented depression (18, 19).

As part of the wider Health Outcomes and Lifestyle In a Sample of people with Multiple Sclerosis (HOLISM) Study, we previously described the results from our baseline cross-sectional study of 2,466 participants with MS (20), finding 19.3% screened positive for depression using the Patient Health Questionnaire-2 (21). We demonstrated strong and clinically meaningful associations between modifiable lifestyle risk factors and depression prevalence. In our current study, we investigated whether modifiable lifestyle factors were associated with screening positive for depression 2.5 years after our baseline study and the predictors of change in depression screen during follow-up.

## Methods

### Participants and recruitment

The methodology for the HOLISM study has previously been documented in detail at both baseline and 2.5-years follow-up (22, 23). Briefly, participants were recruited via online platforms that engaged people with MS. Participants used SurveyMonkey® to complete the survey and to provide informed consent. Participants were eligible if they reported having been diagnosed with MS by a medical doctor and were over 18 years old. Ethics approval was granted by St Vincent's Hospital Melbourne HREC initially, and the Health Sciences Human Ethics Subcommittee at the University of Melbourne (Ethics ID: 1545102).

### Data collection and tools used

Many of the same measures used at baseline were employed at follow-up to allow longitudinal analysis. A range of sociodemographic, behavioral/environmental, and clinical parameters were queried by participant-completed questionnaires (22). Particular elements queried are described below.

### Sociodemographic and biometric data

Data were collected on sex, age, height, weight, country of birth and residence, marital status, education level, employment and socioeconomic status among others.

### Dietary habits

We used a modified version of the Diet Habits Questionnaire (DHQ) (24) as previously described (22, 23). A higher score indicated a healthier diet and data were grouped into quartiles of their total score.

### Vitamin D supplementation

Participants were asked if they took a vitamin D supplement, the amount taken, frequency and duration of supplementation (22).

### Omega-3 supplementation

We assessed both the type and dose of omega-3 supplementation used by participants (22).

### Exercise

We used the International Physical Activity Questionnaire-Short Form (IPAQ-SF) (25), which assesses the frequency and duration of moderate and vigorous physical activity over the preceding 7 days. Data were categorized as low, moderate or high activity level according to the IPAQ guidelines.

### Meditation

We assessed how often participants meditated on average per week and for how long each time (22).

### Alcohol

We asked participants the frequency and volume of alcohol consumed, providing participants information of what a standard drink was. Data was then re-calculated in grams of alcohol to derive variables of low, moderate and high alcohol intake (26).

### Smoking

Participants smoking behavior was queried and these then classified as being a never smoker, ex-smoker or current smoker.

### Depression

At 2.5-years follow-up, we used the Patient Health Questionnaire-9 (PHQ-9) to assess depression risk (27). The PHQ-9 is a nine-question instrument that is widely used and has been validated in MS research (28). The PHQ-2 was used at baseline and includes two items of the PHQ-9, allowing calculation of PHQ-2 and PHQ-9 scores at follow-up review, and thus change in PHQ-2 between reviews. We used the PHQ-9 at follow-up due to its superior psychometric assessment to substantiate findings from baseline.

Participants were asked the frequency of the specific symptom in the past 2 weeks, with answers including “Not at all,” “Several days,” “More than half the days,” and “Nearly every day.” At least one of the asterixed two symptoms must be present for diagnosis of a Major Depressive Episode (29) and these items are included in the PHQ-2.

1) Little interest or pleasure in doing things^*^
2) Feeling down, depressed or hopeless^*^
3) Trouble falling asleep or staying asleep, or sleeping too much
4) Feeling tired or having little energy
5) Poor appetite or overeating
6) Feeling bad about yourself–or that you are a failure or having let yourself or your family down
7) Trouble concentrating on things, such as reading the newspaper or watching television
8) Moving or speaking so slowly that other people could have noticed; or the opposite, being so fidgety or restless that you have been moving around a lot more than usual
9) Thoughts that you would be better off dead or hurting yourself in some way.

The PHQ-2 score ranges from 0 to 6 with scores >2 indicating a positive depression screen. The PHQ-9 score ranges 0–27, with scores >9 indicating positive depression-screen. Additionally, the PHQ-9 can be subdivided into grades of severity: 5–9 indicating minimal depression, 10–14 indicating mild depression, 15–19 indicating moderate depression, and 20–27 indicating severe depression. Moderate and severe depression were combined due to cell-size constraints (*n* = 37 with severe depression).

### Clinical measures

Disability was assessed using the Patient-Determined Disease Steps (PDDS) scale (30), from which the disease-duration adjusted Patient-derived Multiple Sclerosis Severity Score (P-MSSS) was calculated (31). Fatigue was assessed using the Fatigue Severity Scale (FSS) (32). Immunomodulatory medication use, including interferon-beta-based medication, glatiramer acetate, alemtuzumab, cladribine, daclizumab, dimethyl fumarate, fingolimod, laquinimod, rituximab, teriflunomide, and natalizumab, as well as prescription antidepressant and anxiolytic medication use were queried at each review.

### Data analysis

Log-binomial regression models were used to evaluate associations of sociodemographic and lifestyle factors with positive depression-screen at follow-up, estimating a prevalence ratio. Log-multinomial regression models (33) were used to evaluate predictors of severity of PHQ-9 positive depression-screen. Multivariable models at 2.5-years follow-up were adjusted for contemporaneous P-MSSS, age, fatigue, and antidepressant medication use, these covariates were selected based on review of the literature for relevant characteristics and on material impact on models.

Log-binomial regression were used to evaluate sociodemographic and lifestyle factors associated with change in PHQ-2-defined depression-screen between baseline and 2.5-years follow-up. We estimated a risk ratio for baseline predictors and a prevalence ratio where change in determinants was evaluated against change in depression-screen state. In these data analyses, those who changed from positive to negative depression-screen were compared to those who screened positive for depression at both timepoints, while those who changed from negative to positive depression-screen were compared to those who screened negative for depression at both timepoints. Multivariable models for predictors of change in depression-screen were adjusted for P-MSSS, age, fatigue, antidepressant medication use, and baseline continuous PHQ-2 score, these covariates were selected based on review of the literature for relevant characteristics and on material impact on models.

All multivariable models were done using complete-case analysis, that is they were constrained to those who had data on all the model covariates.

STATA/SE 15.0 (StataCorp, College Park, TX, USA) was used to analyse the data as previously described.

## Results

At baseline review, 2,466 participants with MS initiated the questionnaire, of whom 2,224 (88.9%) completed the PHQ-2 instrument. At 2.5-years follow-up review, 1,401 participants with MS initiated the questionnaire, of whom 1,309 (93.4%) completed the PHQ-2 instrument and 1,264 (90.2%) completed the PHQ-9 instrument. The prevalence of depression at 2.5-years review differed between the PHQ-2 and PHQ-9, the PHQ-2 estimating a prevalence of 14.5%, while the PHQ-9 estimated a prevalence of 21.7%.

As described elsewhere (22, 23, 26) the cohort was largely female at both timepoints, of mean age in the mid-40s, and, while the mean BMI was in the overweight range, the cohort consistently engaged in healthy behaviors, including >90% non-smoking, over half engaging in regular physical activity, and large proportions reporting vitamin D and omega-3 supplement use. Alcohol consumption was common, only around 20% reporting not drinking alcohol at either timepoint, though of those using alcohol, the majority drank low/moderate amounts. Diet quality scores were good, with average scores of 81 at both timepoints, particularly driven by high sub-scores in not having snacks and takeaway, lower fat consumption, and healthier food choices (data not shown). Other cohort characteristics are shown in Table 1.

**Table 1:** Cohort characteristics at baseline and 2.5-yr follow-up, and characteristics of those retained at 2.5-years review vs. those lost to follow-up.

	**Baseline** **(*n* = 2,466)**	**Baseline, completed 2.5-years** **(*n* = 1,401)**	**2.5-years** **(*n* = 1,401)**
**PHQ-2 SCORE** > **2**			
No depression risk	1,799 (80.9%)	1,139 (86.3%)	1,119 (85.5%)
Depression risk	425 (19.1%)	181 (13.7%)	190 (14.5%)^‡^
(Missing)	(242 (9.8%))	(81 (5.8%))	(92 (6.6%))^‡^
**PHQ-9 SCORE**			
0–4: normal			607 (48.0%)
5–9: minimal depression symptoms			383 (30.3%)
10–14: major depression, mild			144 (11.4%)
15–19: major depression, moderate			93 (7.4%)
≥20: major depression, severe			37 (2.9%)
(Missing)			(137 (9.8%))
**PHQ-9 SCORE** > **9**			
No depression risk			990 (78.3%)
Depression risk			274 (21.7%)
(Missing)			(137 (9.8%))
**REGION OF RESIDENCE**			
Australasia	835 (34.0%)	560 (40.1%)	564 (40.3%)
Europe	648 (26.4%)	380 (27.2%)	378 (27.0%)
North America	913 (37.1%)	426 (30.5%)	430 (30.7%)^‡^
Other	63 (2.6%)	30 (2.2%)	29 (2.1%)
(Missing)	(7 (0.3%))	(5 (0.4%))	(0 (0%))
**SEX**			
Male	415 (17.6%)	241 (17.3%)	241 (17.3%)
Female	1,937 (82.4%)	1,150 (82.7%)	1,150 (82.7%)
(Missing)	(114 (4.6%))	(10 (0.7%))	(10 (0.7%))^‡^
**SMOKE TOBACCO?**			
Never	1,099 (48.0%)	707 (52.7%)	701 (52.7%)
Ex-smoker	908 (39.7%)	520 (38.8%)	527 (39.6%)
Current smoker	281 (12.3%)	114 (8.5%)	102 (7.7%)^‡^
(Missing)	(178 (7.2%))	(60 (4.3%))	(71 (5.1%))
**ALCOHOL INTAKE**			
Non-drinker	415 (18.2%)	215 (16.1%)	263 (20.7%)
< Once per week	897 (39.3%)	500 (37.3%)	411 (32.4%)^†^^*b*^
1–3 days per week	567 (24.8%)	362 (27.0%)	347 (27.3%)^a^
4–7 days per week	406 (17.8%)	265 (19.8%)	249 (19.6%)^a^
(Missing)	(181 (7.3%))	(59 (4.2%))	(131 (9.4%))^a^
**ALCOHOL LOAD, STANDARD DRINKS PER DAY**^c^			
Low	882 (41.3%)	461 (36.2%)	102 (9.5%)
Moderate	970 (45.4%)	631 (49.6%)	812 (75.8%)^‡^^*b*^
High	286 (13.4%)	180 (14.2%)	157 (14.7%)^‡^^*b*^
(Missing)	(328 (13.3%))	(129 (9.2%))	(330 (23.6%))^‡^^*b*^
**PHYSICAL ACTIVITY**			
Low activity	752 (36.2%)	423 (34.0%)	396 (31.8%)
Moderate activity	839 (40.4%)	533 (42.8%)	582 (46.7%)^†^
High activity	485 (23.4%)	290 (23.3%)	269 (21.6%)
(Missing)	(390 (15.8%))	(155 (11.1%))	(154 (11.0%))^†^
**DIET–CONSUMES MEAT?**			
No	761 (33.2%)	532 (39.6%)	513 (38.5%)
Yes	1,533 (66.8%)	813 (60.5%)	820 (61.5%)^†^
(Missing)	(172 (7.0%))	(56 (4.0%))	(68 (4.9%))^†^
**DIET–CONSUMES DAIRY?**			
No	862 (37.9%)	580 (43.4%)	564 (42.4%)
Yes	1,415 (62.1%)	756 (56.6%)	765 (57.6%)^†^
(Missing)	(189 (7.7%))	(65 (4.6%))	(72 (5.1%))^‡^
**TAKING A VITAMIN D SUPPLEMENT?**			
No	601 (24.4%)	271 (19.3%)	271 (19.3%)
Yes	1,865 (75.6%)	1,130 (80.7%)	1,130 (80.7%)^‡^
**TAKING AN OMEGA-3 SUPPLEMENT?**			
No	998 (40.5%)	469 (33.5%)	542 (38.7%)
Yes	1,468 (59.5%)	932 (66.5%)	859 (61.3%)^a^
**TYPE OF MS AT COMPLETION OF SURVEY**			
Benign	100 (4.1%)	64 (4.6%)	85 (6.2%)^†^^*a*^
RRMS	1,491 (61.6%)	875 (63.3%)	810 (59.2%)
SPMS	275 (11.4%)	144 (10.4%)	199 (14.6%)^†^^*a*^
PPMS	175 (7.2%)	100 (7.2%)	111 (8.1%)
PRMS	48 (2.0%)	18 (1.3%)	23 (1.7%)
Unsure/other	330 (13.6%)	181 (13.1%)	140 (10.2%)^†^
(Missing)	(47 (1.9%))	(19 (1.4%))	(33 (2.4%))^a^
**TAKING ANY OF THE 11 SPECIFIED**			
**IMMUNOMODULATORY MEDICATIONS?**			
No	1,321 (53.6%)	747 (53.3%)	812 (58.0%)
Yes	1,145 (46.4%)	654 (46.7%)	589 (42.0%)^†^^*a*^
**TAKING PRESCRIPTION ANTIDEPRESSANT MEDICATION?**			
No	1,964 (79.6%)	1,158 (82.7%)	1,149 (82.0%)
Yes	502 (20.4%)	243 (17.3%)	252 (18.0%)
**TAKING PRESCRIPTION ANXIOLYTIC MEDICATION?**			
No	2,211 (89.7%)	1,282 (91.5%)	1,285 (91.7%)
Yes	255 (10.3%)	119 (8.5%)	116 (8.3%)^†^
**MEDITATES AT LEAST WEEKLY?**			
No	1,566 (69.8%)	893 (67.2%)	850 (65.0%)
Yes	677 (30.2%)	436 (32.8%)	457 (35.0%)^†^
(Missing)	(223 (9.0%))	(72 (5.1%))	(94 (6.7%))
	**Mean (SD; range)**		
Age	45.7 (10.5; 17.5–79.0)	45.9 (10.5; 17.9–79.0)	48.4^‡^^*b*^ (10.5; 19.3–81.5)
BMI	25.8 (6.5; 14.6–71.0)	25.2 (5.9; 15.4–57.7)	25.4 (6.0; 14.4–64.1)
	**Median (interquartile range)**		
PHQ-2	0 (0–1)	1 (0–2)	0^‡^ (0–1)
PHQ-9			0 (0–0)
IPAQ MET mins per week	1,092 (297–2,826)	1,200 (396–2,826)	1,200 (396–2,670)
DHQ total score	81 (71–89.5)	83 (73.5–91)	81^a^ (71–90)
Disease duration since symptom onset, years	11.8 (6.2–20.4)	11.4 (5.4–20.2)	14.2^‡^^*b*^ (8.1–23.2)
PDDS	2 (0–4)	1 (0–4)	1 (0–4)
P-MSSS	4.7 (2.6–7.4)	4.4 (2.4–7.3)	4.9^a^ (2.6–7.3)
Fatigue Severity Score	44 (29–55)	42 (27–54)	42^†^ (26–54)

*Differences between categorical variables assessed by multinomial logistic regression. Differences between normally distributed continuous terms assessed by two-tailed t-test. Differences between non-normally distributed continuous terms assessed by Kruskal-Wallis rank test*.

† *p < 0.05 for differences between baseline and 2.5-years review*.

‡ *p < 0.001 for differences between baseline and 2.5-years review*.

a *p < 0.05 for differences between baseline and 2.5-years review for participants with 2.5-years follow-up data*.

b *p < 0.001 for differences between baseline and 2.5-years review for participants with 2.5-years follow-up data*.

c *Alcohol intake was categorized specific to sex, such that low alcohol intake was defined as < 15 grams of alcohol per week, moderate was up to 30 grams alcohol per day for females and up to 45 grams alcohol per day for males, and heavy was over 30 grams alcohol per day for females and over 45 grams alcohol per day for males*.

*Note: some variables have missing values but where there were no missing values, this row is not shown for that variable*.

*BMI, body mass index; DHQ, Dietary Habits Questionnaire; IPAQ, International Physical Activity Questionnaire; PDDS, Patient-Determined Disease Steps Scale; PHQ, Patient Health Questionnaire; P-MSSS, Patient Determined Multiple Sclerosis Severity Score; PPMS, primary progressive multiple sclerosis; PRMS, progressive-relapsing multiple sclerosis; RRMS, relapsing-remitting multiple sclerosis; SCQ, Self-administered Comorbidity Questionnaire; SPMS, secondary progressive multiple sclerosis*.

### Determinants of depression at 2.5-years follow-up

Current smokers were significantly more likely to have scores indicative of prevalent depression risk, both PHQ-2 and PHQ-9 (Table 2). Alcohol, on the other hand, showed a significant inverse association with depression risk, showing evidence of a dose-dependent association, particularly PHQ-9. Examining alcohol load found this association was solely driven by low/moderate consumption, with high alcohol consumption not significantly associated with depression risk (data not shown).

**Table 2:** Predictors of depression risk at 2.5-years follow-up.

	**PHQ-2**			**PHQ-9**		
	**n/N with PHQ-2 > 2 (%)**	**Univariable**	**Adjusted**	**N with PHQ-9 > 9 (%)**	**Univariable**	**Adjusted**
**SMOKE TOBACCO?**						
Never	84/695 (12.1%)	1.00 [Reference]	1.00 [Reference]	121/677 (17.9%)	1.00 [Reference]	1.00 [Reference]
Ex-smoker	78/513 (15.2%)	1.26 (0.95, 1.68)	1.20 (0.91, 1.58)	107/491 (21.8%)	1.22 (0.97, 1.54)	1.13 (0.90, 1.41)
Current smoker	28/99 (28.35)	**2.34 (1.61, 3.40)**	**1.63 (1.12, 2.37)**	46/94 (48.9%)	**2.74 (2.11, 3.56)**	**1.96 (1.51, 2.55)**
*Trend:*		***p**<**0.001***	***p** = **0.016***		***p**<**0.001***	***p**<**0.001***
**ALCOHOL INTAKE**						
Non-drinker	45/256 (17.6%)	1.00 [Reference]	1.00 [Reference]	69/246 (28.1%)	1.00 [Reference]	1.00 [Reference]
< Once per week	69/405 (17.0%)	0.97 (0.69, 1.36)	1.04 (0.74, 1.45)	98/387 (25.3%)	0.90 (0.69, 1.18)	0.89 (0.69, 1.15)
1–3 days per week	41/342 (12.0%)	0.68 (0.46, 1.01)	0.82 (0.55, 1.20)	59/333 (17.7%)	**0.63 (0.47, 0.86)**	**0.74 (0.55, 0.99)**
4–7 days per week	27/245 (11.0%)	**0.63 (0.40, 0.98)**	0.83 (0.54, 1.27)	39/240 (16.3%)	**0.58 (0.41, 0.82)**	0.74 (0.53, 1.03)
(Missing)		***p** = **0.007***	*p = 0.18*		***p**<**0.001***	***p** = **0.030***
**ALCOHOL LOAD**^a^						
Low	21/102 (20.6%)	1.00 [Reference]	1.00 [Reference]	29/97 (29.9%)	1.00 [Reference]	1.00 [Reference]
Moderate	109/799 (13.6%)	0.66 (0.44, 1.01)	0.80 (0.53, 1.23)	151/775 (19.5%)	**0.65 (0.47, 0.91)**	0.77 (0.56, 1.06)
High	21/156 (13.5%)	0.65 (0.38, 1.14)	0.84 (0.48, 1.45)	34/154 (22.1%)	0.74 (0.48, 1.13)	0.97 (0.65, 1.45)
*Trend:*		*p = 0.19*	*p = 0.52*		*p = 0.32*	*p = 0.98*
**DHQ TOTAL SCORE**						
32–70	74/336 (22.0%)	1.00 [Reference]	1.00 [Reference]	111/323 (34.4%)	1.00 [Reference]	1.00 [Reference]
>70–80	51/320 (15.9%)	**0.72 (0.52, 1.00)**	0.80 (0.58, 1.11)	83/309 (26.9%)	**0.78 (0.62, 0.99)**	0.87 (0.69, 1.10)
>80–89	46/349 (13.2%)	**0.60 (0.43, 0.84)**	0.82 (0.59, 1.14)	58/335 (17.3%)	**0.50 (0.38, 0.67)**	**0.67 (0.51, 0.88)**
>89–100	19/303 (6.3%)	**0.29 (0.18, 0.46)**	**0.50 (0.31, 0.80)**	22/296 (7.4%)	**0.22 (0.14, 0.33)**	**0.36 (0.24, 0.55)**
*Trend:*		***p**<**0.001***	***p** = **0.005***		***p**<**0.001***	***p**<**0.001***
**CONSUMES MEAT?**						
No	53/505 (10.5%)	1.00 [Reference]	1.00 [Reference]	70/490 (14.3%)	1.00 [Reference]	1.00 [Reference]
Yes	137/803 (17.1%)	**1.63 (1.21, 2.19)**	1.22 (0.92, 1.63)	204/773 (26.4%)	**1.85 (1.44, 2.37)**	**1.41 (1.11, 1.78)**
		***p** = **0.001***	*p = 0.17*		***p**<**0.001***	***p** = **0.004***
**CONSUMES DAIRY?**						
No	58/551 (10.5%)	1.00 [Reference]	1.00 [Reference]	71/534 (13.3%)	1.00 [Reference]	1.00 [Reference]
Yes	131/753 (17.4%)	**1.65 (1.24, 2.21)**	1.27 (0.96, 1.69)	203/725 (28.0%)	**2.11 (1.65, 2.69)**	**1.60 (1.26, 2.02)**
		***p** = **0.001***	*p = 0.10*		***p**<**0.001***	***p**<**0.001***
**TAKING A VITAMIN D SUPPLEMENT?**						
No	49/192 (25.5%)	1.00 [Reference]	1.00 [Reference]	63/186 (33.9%)	1.00 [Reference]	1.00 [Reference]
Yes	141/1,117 (12.6%)	**0.50 (0.37, 0.66)**	**0.61 (0.46, 0.81)**	211/1,078 (19.6%)	**0.58 (0.46, 0.73)**	**0.70 (0.56, 0.87)**
		***p**<**0.001***	***p** = **0.001***		***p**<**0.001***	***p** = **0.002***
**TALKING AN OMEGA-3 SUPPLEMENT?**						
No	87/464 (18.8%)	1.00 [Reference]	1.00 [Reference]	128/450 (28.4%)	1.00 [Reference]	1.00 [Reference]
Yes	103/845 (12.2%)	**0.65 (0.50, 0.85)**	0.87 (0.67, 1.12)	146/814 (17.9%)	**0.63 (0.51, 0.78)**	**0.80 (0.66, 0.98)**
		***p** = **0.001***	*p = 0.28*		***p**<**0.001***	***p** = **0.031***
**PHYSICAL ACTIVITY, BY IPAQ**						
Low activity	86/391 (22.0%)	1.00 [Reference]	1.00 [Reference]	122/377 (32.4%)	1.00 [Reference]	1.00 [Reference]
Moderate activity	69/577 (12.0%)	**0.54 (0.41, 0.73)**	0.75 (0.55, 1.01)	97/556 (17.5%)	**0.54 (0.43, 0.68)**	**0.74 (0.59, 0.93)**
High activity	16/268 (6.0%)	**0.27 (0.16, 0.45)**	**0.49 (0.30, 0.82)**	30/261 (11.5%)	**0.36 (0.25, 0.51)**	**0.60 (0.42, 0.87)**
*Trend:*		***p**<**0.001***	***p** = **0.003***		***p**<**0.001***	***p** = **0.002***
**MEDITATES AT LEAST WEEKLY?**						
No	133/839 (15.9%)	1.00 [Reference]	1.00 [Reference]	198/817 (24.2%)	1.00 [Reference]	1.00 [Reference]
Yes	50/454 (11.0%)	**0.70 (0.51, 0.94)**	0.75 (0.56, 1.00)	70/432 (16.2%)	**0.67 (0.52, 0.86)**	**0.73 (0.58, 0.92)**
		***p** = **0.019***	*p = 0.052*		***p** = **0.001***	***p** = **0.008***

*Analyses by log-binomial regression, estimating a prevalence ratio (PR) (95% CI). Adjusted models adjusted for age, P-MSSS, FSS, and use of antidepressant medication*.

*Figures in boldface denote statistical significance (p < 0.05). Figures in italics are p-values*.

a *Alcohol intake was categorized specific to sex, such that low alcohol intake was defined as < 15 grams of alcohol per week, moderate was up to 30 grams alcohol per day for females and up to 45 grams alcohol per day for males, and heavy was over 30 grams alcohol per day for females and over 45 grams alcohol per day for males*.

*BMI, body mass index; DHQ, Dietary Habits Questionnaire; FSS, Fatigue Severity Scale; IPAQ, International Physical Activity Questionnaire; PHQ, Patient Health Questionnaire; P-MSSS, Patient Determined Multiple Sclerosis Severity Score*.

Higher diet quality scores also showed a dose-dependent association with lower frequencies of depression, though adjustment attenuated these associations. Those reporting consuming meat and dairy had higher frequencies of depression at follow-up, especially the PHQ-9, though adjustment attenuated all these associations. Both vitamin D and omega-3 supplementation were associated with lower frequencies of depression risk by both PHQ-2 and PHQ-9. However, while the associations of vitamin D supplementation persisted on adjustment, omega-3 associations were greatly attenuated.

Greater physical activity was associated with a significantly lower depression risk, robust to adjustment. Likewise, meditation was associated with significantly reduced prevalence of depression by both scores, though only that for PHQ-9 persisted on adjustment.

### Determinants of PHQ-9 grade of depression at 2.5-years follow-up

Many of the factors associated with overall depression risk (PHQ-9 > 9) in Table 2 were also associated with the gradations of depression severity (Table 3). The positive associations of current smoking with depression risk were much stronger for major depression. There was an inverse association between alcohol and overall depression risk, only evident for major depression, while no associations were seen for minimal depression symptoms. As with overall depression risk, however, while both moderate and high alcohol intake were inversely associated with major depression risk, there was no benefit of high alcohol intake with depression (data not shown).

**Table 3:** Predictors of depression risk level vs. no depression risk at 2.5-years follow-up, as measured by PHQ-9.

	**N with PHQ9 =** **0–4 (Normal)** **(%)**	**N with PHQ9 =** **5–9 (Minimal)** **(%)**	**N with PHQ9 =** **10–14 (Major, moderate/severe)** **(%)**	**N with PHQ9 = ≥15 (Major, moderate/severe)** **(%)**	**aPR Minimal depression symptoms vs. no depression risk**	**aPR Major depression, mild vs. no depression risk**	**aPR Major depression, moderate/severe vs. no depression risk**
**SMOKE TOBACCO?**							
Never	358 (52.9%)	198 (29.3%)	67 (9.9%)	54 (8.0%)	1.00 [Reference]	1.00 [Reference]	1.00 [Reference]
Ex-smoker	228 (46.4%)	156 (31.8%)	54 (11.0%)	53 (10.8%)	1.06 (0.91, 1.23)	1.04 (0.77, 1.41)	1.33 (0.99, 1.79)
Current smoker	21 (22.3%)	27 (28.7%)	23 (24.5%)	23 (24.5%)	**1.34 (1.02, 1.75)**	**2.17 (1.50, 3.15)**	**1.99 (1.45, 2.74)**
*Trend:*					*p = 0.071*	***p** = **0.002***	***p**<**0.001***
**ALCOHOL INTAKE**							
Non-drinker	107 (43.5%)	70 (28.5%)	32 (13.0%)	37 (15.0%)	1.00 [Reference]	1.00 [Reference]	1.00 [Reference]
< Once per week	170 (43.9%)	119 (30.8%)	53 (13.7%)	45 (11.6%)	1.03 (0.84, 1.26)	0.98 (0.69, 1.40)	0.78 (0.57, 1.06)
1–3 days per week	174 (52.3%)	100 (30.0%)	32 (9.6%)	27 (8.1%)	0.97 (0.78, 1.20)	0.74 (0.49, 1.11)	**0.63 (0.42, 0.94)**
4–7 days per week	130 (54.2%)	71 (29.6%)	24 (10.0%)	15 (6.3%)	0.97 (0.78, 1.22)	0.80 (0.53, 1.22)	0.68 (0.41, 1.11)
*Trend:*					*p = 0.67*	*p = 0.13*	***p** = **0.030***
**ALCOHOL LOAD**^a^							
Low	41 (42.3%)	27 (27.8%)	11 (11.3%)	18 (18.6%)	1.00 [Reference]	1.00 [Reference]	1.00 [Reference]
Moderate	388 (50.1%)	236 (30.5%)	82 (10.6%)	69 (8.9%)	1.00 (0.76, 1.31)	0.88 (0.52, 1.48)	**0.62 (0.43, 0.89)**
High	74 (48.1%)	46 (29.9%)	23 (14.9%)	11 (7.1%)	1.09 (0.79, 1.51)	1.29 (0.73, 2.29)	0.75 (0.41, 1.38)
*Trend:*					*p = 0.48*	*p = 0.14*	*p = 0.18*
**DHQ TOTAL SCORE**							
32–70	104 (32.3%)	107 (33.2%)	52 (16.2%)	59 (18.3%)	1.00 [Reference]	1.00 [Reference]	1.00 [Reference]
>70–80	129 (41.8%)	97 (31.4%)	45 (14.6%)	38 (12.3%)	0.88 (0.73, 1.07	0.81 (0.60, 1.11)	0.79 (0.58, 1.09)
>80–89	178 (53.1%)	99 (29.6%)	34 (10.2%)	24 (7.2%)	**0.77 (0.63, 0.93)**	**0.67 (0.47, 0.96)**	**0.60 (0.42, 0.87)**
>89–100	195 (65.9%)	79 (26.7%)	13 (4.4%)	9 (3.0%)	**0.70 (0.57, 0.87)**	**0.33 (0.19, 0.58)**	**0.33 (0.17, 0.62)**
*Trend:*					***p**<**0.001***	***p**<**0.001***	***p**<**0.001***
**CONSUMES MEAT?**							
No	290 (59.2%)	130 (26.5%)	42 (8.6%)	28 (5.7%)	1.00 [Reference]	1.00 [Reference]	1.00 [Reference]
Yes	317 (41.0%)	252 (32.6%)	102 (13.2%)	102 (13.2%)	**1.28 (1.09, 1.49)**	**1.41 (1.04, 1.90)**	**1.51 (1.07, 2.13)**
					***p** = **0.002***	***p** = **0.026***	***p** = **0.020***
**CONSUMES DAIRY?**							
No	311 (58.2%)	152 (28.5%)	39 (7.3%)	32 (6.0%)	1.00 [Reference]	1.00 [Reference]	1.00 [Reference]
Yes	293 (40.4%)	229 (31.6%)	105 (14.5%)	98 (13.5%)	**1.21 (1.04, 1.40)**	**1.61 (1.18, 2.21)**	**1.49 (1.06, 2.10)**
					***p** = **0.012***	***p** = **0.003***	***p** = **0.022***
**TAKING A VITAMIN D SUPPLEMENT?**							
No	66 (35.5%)	57 (30.7%)	28 (15.1%)	35 (18.8%)	1.00 [Reference]	1.00 [Reference]	1.00 [Reference]
Yes	541 (50.2%)	326 (30.25)	116 (10.8%)	95 (8.8%)	0.91 (0.76, 1.10)	0.73 (0.53, 1.00)	**0.61 (0.46, 0.79)**
					*p = 0.35*	*p = 0.050*	***p**<**0.001***
**TALKING AN OMEGA-3 SUPPLEMENT?**							
No	177 (39.3%)	145 (32.2%)	69 (15.3%)	59 (13.1%)	1.00 [Reference]	1.00 [Reference]	1.00 [Reference]
Yes	430 (52.8%)	238 (29.2%)	75 (9.2%)	71 (8.7%)	0.91 (0.79, 1.05)	**0.75 (0.57, 0.99)**	0.84 (0.65, 1.09)
					*p = 0.19*	***p** = **0.039***	*p = 0.18*
**PHYSICAL ACTIVITY**							
Low activity	127 (33.7%)	128 (34.0%)	64 (17.0%)	58 (15.4%)	1.00 [Reference]	1.00 [Reference]	1.00 [Reference]
Moderate activity	294 (52.9%)	165 (29.7%)	53 (9.5%)	44 (7.9%)	0.93 (0.79, 1.10)	**0.72 (0.53, 0.98)**	0.72 (0.52, 1.00)
High activity	162 (62.1%)	69 (26.4%)	20 (7.7%)	10 (3.8%)	0.96 (0.77, 1.20)	0.69 (0.44, 1.10)	**0.48 (0.27, 0.86)**
*Trend:*					*p = 0.57*	***p** = **0.035***	***p** = **0.004***
**MODERATE/HIGH PHYSICAL ACTIVITY?**							
No	127 (33.7%)	128 (34.0%)	64 (17.0%)	58 (15.4%)	1.00 [Reference]	1.00 [Reference]	1.00 [Reference]
Yes	456 (55.8%)	234 (28.6%)	73 (8.9%)	54 (6.6%)	0.94 (0.80, 1.10)	**0.71 (0.53, 0.96)**	**0.67 (0.49, 0.91)**
					*p = 0.44*	***p** = **0.024***	***p** = **0.012***
**MEDITATES AT LEAST WEEKLY?**							
No	374 (45.8%)	245 (30.0%)	109 (13.3%)	89 (10.9%)	1.00 [Reference]	1.00 [Reference]	1.00 [Reference]
Yes	229 (53.0%)	133 (30.8%)	35 (8.1%)	35 (8.1%)	0.99 (0.85, 1.15)	**0.66 (0.48, 0.90)**	0.85 (0.63, 1.14)
					*p = 0.86*	***p** = **0.008***	*p = 0.28*

*Analyses by log-multinomial regression^29^, estimating a prevalence ratio (PR) (95% CI). All models adjusted for age, P-MSSS, FSS, and use of antidepressant medication*.

*Figures in boldface denote statistical significance (p < 0.05). Figures in italics are p-values*.

a *Alcohol intake was categorized specific to sex, such that low alcohol intake was defined as < 15 grams of alcohol per week, moderate was up to 30 grams alcohol per day for females and up to 45 grams alcohol per day for males, and heavy was over 30 grams alcohol per day for females and over 45 grams alcohol per day for males*.

*BMI, body mass index; DHQ, Dietary Habits Questionnaire; FSS, Fatigue Severity Scale; IPAQ, International Physical Activity Questionnaire; PHQ, Patient Health Questionnaire; P-MSSS, Patient Determined Multiple Sclerosis Severity Score*.

Higher diet quality was associated with a significantly reduced risk of depression, most strongly with major depression. Likewise, for meat and dairy consumption, the positive associations were much stronger with severe depression. Vitamin D and omega-3 supplementation both showed strong and significant inverse associations with major depression risk.

The association of physical activity with depression was only evident for major depression, showing no associations with minimal depression symptoms. For major depression, however, those engaging in more physical activity had markedly lower frequencies of major depression, over 50%. Meditation, on the other hand, while strongly associated with PHQ-9 overall, showed no material dose-dependency, only being significantly associated with mild major depression.

### Baseline and trajectory determinants of change in PHQ-2 depression between baseline and 2.5-years follow-up

A change in PHQ-2 depression was evaluated as a change in state between baseline and 2.5-years follow-up, such that participants could go from having not screened positive for depression at baseline to positive screen at follow-up (“becoming depressed”), screening positive for depression at baseline and losing this at follow-up (“losing depression”), or having no change, this including screening positive for depression or not at both timepoints (Table 4). For trajectory analysis, then, gaining depression risk was compared against those without depression risk at both time points, while losing depression risk was compared against those with depression risk at both time points.

**Table 4:** Baseline demographic and lifestyle predictors of change in PHQ-2 depression state between baseline and 2.5-years follow-up.

	**N with depression at both baseline and follow-up (row %)**	**N with depression at baseline, not at follow-up (row %)**	**RR loss of depression vs. always depressed**	**aRR loss of depression vs. always depressed**	**N with negative depression-screen at both baseline and follow-up (row %)**	**N with no depression at baseline but with depression at follow-up (row %)**	**RR gain of depression vs. never depressed**	**aRR gain of depression vs. never depressed**
**SMOKE TOBACCO?**								
Never	23 (35.9%)	41 (64.1%)	1.00 [Reference]	1.00 [Reference]	546 (91.2%)	53 (8.9%)	1.00 [Reference]	1.00 [Reference]
Ex-smoker	33 (46.5%)	38 (53.5%)	0.84 (0.63, 1.11)	0.88 (0.66, 1.18)	363 (90.8%)	37 (9.3%)	1.04 0.70, 1.55)	1.11 (0.74, 1.66)
Current smoker	15 (50.0%)	15 (50.0%)	0.78 (0.52,1.16)	0.79 (0.53, 1.19)	61 (82.4%)	13 (17.6%)	**1.83 (1.04, 3.22)**	1.38 (0.72, 2.63)
*Trend:*			*p = 0.16*	*p = 0.21*			*p = 0.14*	*p = 0.36*
**CURRENT SMOKER?**								
No	56 (41.5%)	79 (58.5%)	1.00 [Reference]	1.00 [Reference]	909 (91.0%)	90 (9.0%)	1.00 [Reference]	1.00 [Reference]
Yes	15 (50.0%)	15 (50.0%)	0.85 (0.58, 1.25)	0.85 (0.58, 1.24)	61 (82.4%)	13 (17.6%)	**1.80 (1.05, 3.09)**	1.33 (0.71, 2.48)
			*p = 0.41*	*p = 0.39*			***p** = **0.033***	*p = 0.38*
**ALCOHOL INTAKE**								
Non-drinker	21 (56.8%)	16 (43.2%)	1.00 [Reference]	1.00 [Reference]	137 (87.3%)	20 (12.7%)	1.00 [Reference]	1.00 [Reference]
< Once per week	28 (40.6%)	41 (59.4%)	1.33 (0.88, 202)	1.56 (0.97, 2.49)	353 (89.1%)	43 (10.9%)	0.82 (0.50, 1.33)	0.72 (0.44, 1.18)
1–3 days per week	10 (33.3%)	20 (66.7%)	1.50 (0.96, 2.35)	**1.71 (1.03, 2.84)**	278 (92.7%)	22 (7.3%)	**0.54 (0.31, 0.95)**	0.61 (0.35, 1.08)
4–7 days per week	12 (41.4%)	17 (58.6%)	1.32 (0.81, 2.13)	1.58 (0.92, 2.73)	203 (91.9%)	18 (8.1%)	0.61 (0.34, 1.10)	0.57 (0.31, 1.05)
*Trend:*			*p = 0.21*	*p = 0.073*			***p** = **0.039***	*p = 0.083*
**DRINKS ALCOHOL?**								
No	21 (56.8%)	16 (43.2%)	1.00 [Reference]	1.00 [Reference]	137 (87.3%)	20 (12.7%)	1.00 [Reference]	1.00 [Reference]
Yes	50 (39.1%)	78 (60.9%)	1.37 (0.92, 2.03)	**1.60 (1.02, 2.51)**	834 (91.0%)	83 (9.1%)	0.68 (0.43, 1.06)	0.65 (0.41, 1.03)
			*p = 0.12*	***p** = **0.041***			*p = 0.088*	*p = 0.065*
**ALCOHOL LOAD**^a^								
Low	37 (50.0%)	38 (50.0%)	1.00 [Reference]	1.00 [Reference]	304 (87.0%)	45 (13.0%)	1.00 [Reference]	1.00 [Reference]
Moderate	21 (32.3%)	44 (67.7%)	**1.33 (1.01, 1.75)**	**1.47 (1.10, 1.96)**	480 (92.1%)	41 (7.9%)	**0.60 (0.41, 0.88)**	0.70 (0.47, 1.05)
High	9 (47.4%)	10 (52.6%)	1.05 (0.65, 1.72)	1.12 (0.67, 1.86)	139 (92.7%)	11 (7.3%)	0.54 (0.30, 1.00)	0.54 (0.27, 1.06)
*Trend:*			*p = 0.28*	*p = 0.14*			***p** = **0.010***	***p** = **0.025***
**HIGH ALCOHOL CONSUMPTION**^a^**?**								
No	58 (42.1%)	82 (57.9%)	1.00 [Reference]	1.00 [Reference]	820 (89.9%)	92 (10.1%)	1.00 [Reference]	1.00 [Reference]
Yes	9 (47.4%)	10 (52.6%)	0.92 (0.58, 1.45)	0.91 (0.57, 1.46)	139 (92.7%)	11 (7.3%)	0.71 (0.39, 1.28)	0.64 (0.33, 1.22)
			*p = 0.71*	*p = 0.69*			*p = 0.25*	*p = 0.18*
**DHQ TOTAL SCORE**								
32–70	25 (43.9%)	32 (56.1%)	1.00 [Reference]	1.00 [Reference]	168 (85.3%)	29 (14.7%)	1.00 [Reference]	1.00 [Reference]
>70–80	20 (41.7%)	28 (58.3%)	0.99 (0.71, 1.39)	0.99 (0.71, 1.38)	206 (88.0%)	28 (12.0%)	0.86 (0.54, 1.38)	0.98 (0.59, 1.60)
>80–89	15 (36.6%)	26 (63.4%)	1.12 (0.81, 1.55)	1.04 (0.75, 1.45)	269 (91.8%)	24 (8.2%)	**0.61 (0.37, 0.99)**	0.79 (0.47, 1.31)
>89–100	11 (55.0%)	9 (45.0%)	0.77 (0.45, 1.30)	0.70 (0.41, 1.18)	332 (93.8%)	22 (6.2%)	**0.47 (0.28, 0.79)**	0.59 (0.34, 1.02)
*Trend:*			*p = 0.69*	*p = 0.44*			***p** = **0.001***	***p** = **0.041***
**CONSUMES MEAT?**								
No	21 (51.2%)	20 (48.8%)	1.00 [Reference]	1.00 [Reference]	426 (92.0%)	37 (8.0%)	1.00 [Reference]	1.00 [Reference]
Yes	50 (40.0%)	75 (60.0%)	1.22 (0.87, 1.72)	1.45 (0.98, 2.16)	548 (89.3%)	66 (10.8%)	1.30 (0.89, 1.89)	1.07 (0.72, 1.59)
			*p = 0.25*	*p = 0.063*			*p = 0.18*	*p = 0.73*
**CONSUMES DAIRY?**								
No	20 (45.5%)	24 (54.6%)	1.00 [Reference]	1.00 [Reference]	466 (93.4%)	33 (6.6%)	1.00 [Reference]	1.00 [Reference]
Yes	51 (42.2%)	70 (57.9%)	1.10 (0.81, 1.50)	1.17 (0.84, 1.62)	503 (88.1%)	68 (11.9%)	**1.72 (1.16, 2.54)**	1.41 (0.94, 2.11)
			*p = 0.54*	*p = 0.36*			***p** = **0.007***	*p = 0.096*
**TALKING A VITAMIN D SUPPLEMENT?**								
No	18 (46.2%)	21 (53.9%)	1.00 [Reference]	1.00 [Reference]	130 (84.4%)	24 (15.6%)	1.00 [Reference]	1.00 [Reference]
Yes	53 (41.7%)	74 (58.3%)	1.04 (0.75, 1.45)	1.03 (0.73, 1.45)	845 (91.5%)	79 (8.6%)	**0.60 (0.40, 0.91)**	**0.64 (0.42, 1.00)**
			*p = 0.82*	*p = 0.87*			***p** = **0.015***	***p** = **0.047***
**TALKING AN OMEGA-3 SUPPLEMENT?**								
No	38 (50.0%)	38 (50.0%)	1.00 [Reference]	1.00 [Reference]	267 (89.9%)	30 (10.1%)	1.00 [Reference]	1.00 [Reference]
Yes	33 (36.7%)	57 (63.3%)	1.23 (0.93, 1.62)	1.25 (0.94, 1.66)	708 (90.7%)	73 (9.4%)	0.96 (0.65, 1.42)	1.08 (0.71, 1.66)
			*p = 0.15*	*p = 0.13*			*p = 0.83*	*p = 0.72*
**PHYSICAL ACTIVITY, BY IPAQ**								
Low activity	37 (42.1%)	51 (58.0%)	1.00 [Reference]	1.00 [Reference]	273 (88.1%)	37 (11.9%)	1.00 [Reference]	1.00 [Reference]
Moderate activity	23 (46.0%)	27 (54.0%)	0.94 (0.69, 1.28)	0.89 (0.64, 1.23)	407 (91.5%)	38 (8.5%)	0.76 (0.50, 1.16)	1.13 (0.72, 1.78)
High activity	6 (30.0%)	14 (70.0%)	1.21 (0.88, 1.67)	1.11 (0.78, 1.59)	236 (92.9%)	18 (7.1%)	0.70 (0.41, 1.18)	1.04 (0.58, 1.88)
*Trend:*			*p = 0.51*	*p = 0.69*			*p = 0.15*	*p = 0.92*
**MODERATE/HIGH PHYSICAL ACTIVITY?**								
No	37 (42.1%)	51 (58.0%)	1.00 [Reference]	1.00 [Reference]	273 (88.2%)	37 (11.8%)	1.00 [Reference]	1.00 [Reference]
Yes	29 (41.4%)	41 (58.6%)	1.02 (0.78, 1.32)	0.95 (0.72, 1.27)	643 (91.5%)	56 (8.5%)	0.80 (0.55, 1.17)	1.18 (0.77, 1.81)
			*p = 0.91*	*p = 0.74*			*p = 0.24*	*p = 0.45*
**MEDITATES AT LEAST WEEKLY?**								
No	48 (37.5%)	80 (62.5%)	1.00 [Reference]	1.00 [Reference]	637 (90.2%)	69 (9.8%)	1.00 [Reference]	1.00 [Reference]
Yes	23 (60.5%)	15 (39.5%)	**0.60 (0.40, 0.92)**	**0.61 (0.40, 0.94)**	337 (90.8%)	34 (9.2%)	0.94 (0.63, 1.38)	0.90 (0.60, 1.35)
			***p** = **0.017***	***p** = **0.024***			*p = 0.73*	*p = 0.62*

*Analyses by log-binomial regression, estimating a risk ratio (RR) (95% CI). Adjusted models adjusted for age, baseline P-MSSS, baseline FSS, and baseline use of antidepressant medication*.

*Figures in boldface denote statistical significance (p < 0.05). Figures in italics are p-values*.

a *Alcohol intake was categorized specific to sex, such that low alcohol intake was defined as < 15 grams of alcohol per week, moderate was up to 30 grams alcohol per day for females and up to 45 grams alcohol per day for males, and heavy was over 30 grams alcohol per day for females and over 45 grams alcohol per day for males*.

*BMI, body mass index; DHQ, Dietary Habits Questionnaire; FSS, Fatigue Severity Scale; IPAQ, International Physical Activity Questionnaire; PHQ, Patient Health Questionnaire; P-MSSS, Patient Determined Multiple Sclerosis Severity Score*.

While there was some indication that smoking had a prospective association with subsequently becoming depressed, these associations were essentially abrogated on adjustment. Alcohol consumption, on the other hand, was associated with greater risk of losing depression and lower risk of becoming depressed, much more robust to adjustment. As seen with cross-sectional depression, there was no association of high alcohol consumption with change in depression state, suggesting these associations are particular to moderate alcohol intake. Higher diet quality showed a prospective association with reduced risk of becoming depressed, fairly robust to adjustment. Meat and dairy consumption were inconsistently associated with change in depression, with some indication that consumption was associated with becoming depressed, but these associations largely attenuated on adjustment. Vitamin D supplementation showed a strong association with a reduced risk of becoming depressed, robust to adjustment. Omega-3 supplementation was not associated with either becoming depressed or losing depression.

Overall physical activity was not associated with change in depression state. Of interest, those who reported meditating at least weekly at baseline had a significantly reduced risk of losing depression, robust to adjustment, though there was no association of meditation with risk of gaining depression.

## Discussion

Depression is common (1), poorly treated, under-diagnosed (34) and has been reported to exert the greatest influence on quality of life for people with MS, irrespective of disability level (35). In the general population, depression typically has a strong genetic basis and has episodes with full or partial recovery (29). However, for people with MS, depression is persistent (8) and genetic determinants are not the primary drivers (1). Instead, for people with MS depression is likely due to the psychological adjustment to the illness, as well as underlying physiological processes driving the disease. While it is well-established that the underlying processes of MS are multifactorial, including neurodegeneration, autoimmunity and inflammation; depression has more recently been recognized as having an inflammatory component mediated by modifiable lifestyle factors (16, 36). Lifestyle factors, combined with the stress of diagnosis and adjustment to illness, may cause overactivation of the hypothalamus-pituitary-adrenal axis, increasing cortisol and systemic inflammation (16). Identification of risk factors for MS and depression and their link via the common pathway of inflammation opens the critical avenue for preventive and therapeutic interventions (20, 36) potentially leading to improved morbidity and mortality outcomes.

In the current study, we have completed a comprehensive investigation of modifiable lifestyle factors associated with screening positive for depression, and the predictors of change in depression state over 2.5 years of follow-up. Moderate alcohol use was associated with lower depression risk, particularly severe depression at 2.5-years follow-up, which is consistent with our baseline findings (20). These results are also in keeping with the literature, where moderate alcohol intake has been associated with better mental health-related quality of life in MS (26). In the general population, moderate alcohol intake has been associated with lower rates of depression in primary care settings (37). Beyond the association of alcohol use and depression, we found that moderate alcohol intake was associated with greater risk of losing depression and lower risk of becoming depressed. These data are supported by findings in the general population that moderate alcohol intake is associated with lower incidence of becoming depressed (38). Moderate alcohol intake results in significant reduction of proinflammatory cytokines (39), reducing inflammation, suggesting a mechanism of preventing and potentially treating depression. Heavy alcohol use or alcohol dependence, on the other hand, is harmful for general health and increases the prevalence of depression in both the general population and MS (37, 38, 40). Our HOLISM findings are consistent with this literature, finding no beneficial association of heavy alcohol intake and depression trajectory, nor with mental or physical health benefits (20, 26).

Being a smoker was significantly predictive of positive depression screen and more severe depression at follow-up. This finding parallels the association between smoking and lower mental health-related quality of life, which we previously demonstrated in people with MS (26). After adjusting for potential confounders, we did not find an association between smoking and subsequent change in depression risk, however, while the clinical implications of smoking and depression require further research, the evidence base is clear that smoking is a major risk factor for the development and progression of MS and other comorbidities (41, 42). Smokers commonly have depression, and vice versa, people with depression are more likely to smoke, and smoking cessation interventions are more successful if management includes interventions for depression (43). Being or becoming a non-smoker clearly has multiple direct and indirect benefits for people with MS.

Intervention trials in the general population show that improving diet quality, increasing exercise, sunlight exposure (44), and supplementing with omega-3 and vitamin D are effective adjunctive treatments to antidepressant medication (45). Vitamin D and omega-3 supplementation was common in this sample, with roughly three-quarters taking vitamin D and two-thirds taking omega-3 supplements. This supplement use is becoming quite common among MS patients, given the abundance of research evidence suggesting a potential protective association on MS onset and progression. While not yet proven, and thus not recommended by medical practitioners as a treatment against MS, such supplementation is a relatively inexpensive and simple lifestyle modification for people to undertake, and one without material side effect, and consequently it is frequently seen in MS cohorts.

Observational studies in the general population and MS indicate that low vitamin D is a modifiable risk factor for depression (46, 47). Our data at baseline and 2.5-years follow-up found an association between supplementation with vitamin D and risk of positive depression screen, and as well as severity of depression.

A recent meta-analysis supports that omega-3 intake is associated with a lower risk of depression (48). In line with the literature in the general population, our data found an inverse association between omega-3 supplementation and both the risk of positive depression screen and severity of depression, though not with change in depression state. The literature on health benefits for people with MS is not consistent. While omega-3 supplementation has been associated with better health-related quality of life and reduced disability among people with MS (49), another study found no benefit for disability progression, quality of life, relapses, MRI lesions or fatigue (50). A recent pilot trial found no effect of omega-3 on depression in people with MS (51). However, omega-3 supplementation is safe and provides numerous health benefits across the life cycle and in other diseases, such as cancer and autoimmune conditions (52–54). Its effects are thought to be via immunomodulation, anti-inflammation, neuroprotection and neurotransmission (55). Reverse causality is possible, as people without depression are more likely to engage in more healthy behaviors. Nonetheless, our data and other studies support a possible link between supplementation of omega-3, and mental and physical health outcomes in people with MS (20, 49).

Healthy diet is critical for optimal neurological function (16), evidenced in our growing knowledge of the connection between the gut microbiome, neurotransmitters and mental health (56). While we found no association between diet quality and depression risk, there was a dose-response association between diet quality and depression severity. Dose-response relationships have been found between diet quality and depression in the general population (57) and better diet quality has been associated with improved mental health-related quality of life in people with MS (58). Dairy, but not meat intake, was associated with greater depression risk on PHQ-9 but not PHQ-2. In our study, the relationship between diet and depression risk may reflect reverse causality. The observed association of dairy intake and depression risk is less clear and is likely also to be affected by reverse causality. These results are partly in line with previous work, including work showing a vegan diet improved depression in the general population (59). A whole food plant-based diet has been recommended for people with MS and more widely for general health (60). It is important for clinicians to consider dietary advice as part of an effective management strategy for depression (61).

Low physical activity was strongly associated with an increased depression risk and depression severity. We did, however, not find an association between physical activity and change in depression risk. Our data reinforce findings that people with MS who exercise regularly have better quality of life and favorable depression scores (57, 62) consistent with findings in the general population (63). The relationship between physical activity and depression is likely bi-directional and we cannot quantify the degree of reverse causality that may be present in our data. However, the evidence base from clinical trials is strong enough for clinicians to inform people with MS of the benefit of regular physical activity (64). There is a synergistic benefit to be gained through regular exercise on mood and to reduce obesity and comorbid medical disorders for direct benefit in MS (16, 65, 66).

Meta-analysis of meditation and mindfulness-based stress reduction programs shows a benefit for depression across the general population and for people with other chronic illness (16, 67). For people with MS, mindfulness meditation holds potential to improve immune function and reduce inflammation (68). A recent randomized controlled trial of mindfulness-based interventions improved depression and quality of life, with gains maintained at long-term follow-up (14). In line with these studies, we have shown meditation is cross-sectionally associated with lower depression risk at 2.5-years follow-up, although our findings did not show meditation to be associated with depression trajectory during follow-up. This disparity may reflect the relative insensitivity of our assessment of meditation, as well as the inherent subjectivity of meditation. Thus, further longitudinal analyses, ideally with rigorous assessment of meditation behavior, are needed to assess the role of meditation in depression and MS.

A preventive medical approach to MS management, both pharmacological and otherwise, is in line with the broad shift toward early intervention in the disease course (69). At baseline (20) and here at 2.5-years follow-up, the HOLISM study has shown clinically and statistically significant associations between key modifiable lifestyle risk factors and depression, as well as better mental and physical health-related quality of life (20, 58). Moreover, in another study of people with MS who attended lifestyle modification workshops, we found showed improved mental health-related quality of life at 1, 3 and 5 years follow-up (70–72), also finding that those with greater adherence to lifestyle modification had better outcomes. More complete data revealed adherence to lifestyle changes and outcome improvements at 1 and 3 years follow-up, including stabilized disability, reduced relapse rates, and better physical health-related quality of life (72). These results are supported by work in other chronic illnesses, finding depression was reduced in people with diabetes and elevated coronary risk factors who underwent intensive lifestyle modification (73). Thus, there is potential for lifestyle factors like those assessed here to have positive effects on depression. If validated in other samples and supported by randomized controlled trials, such lifestyle modification could be an additional point of intervention to improve depression among people living with MS.

### Strengths and limitations

A major strength of our sample was the breadth of data and exposure gradient for lifestyle factors and sociodemographics. However, some subsets of data, such as severe depression remained small. It may be that such severely depressed people would not participate in this study and thus, our assessment of the frequency and determinants of severe depression may be affected. Our sample may be biased due to participants being recruited via online platforms, potentially recruiting a healthier and more actively engaged sample of people with MS at baseline and follow-up. In addition, there was appreciable attrition between baseline and follow-up reviews, with a retention rate of 56.8%. While there was some evidence that those retained in the study engaged in more healthy behaviors like not smoking, other behaviors like alcohol, physical activity and supplement use were not materially different between the original sample and those participating at follow-up, nor were clinical characteristics like disability or fatigue materially different. However, significantly more people with depression risk at baseline were lost to follow-up, suggesting that our estimates of depression prevalence at follow-up may underestimate the true prevalence, and that associations with depression state may be affected by this differential loss to follow-up.

Our data are self-reported so the potential for recall bias exists. Reverse causality cannot be excluded from some associations and may have contributed to some of our trajectory data. However, the biological plausibility, dose-response effect and results from existing literature supports a potential causal relationship between several lifestyle factors and depression risk. Our data have many strengths, we recruited and retained a large sample size, including people with all types of MS from geographically diverse backgrounds. Validated tools were used wherever possible and potential confounders were adjusted for. However, not all participants responded to every question and thus, there was some missing data. Accordingly, all multivariate models were complete-case analysis, restricted to those with data on all model parameters.

A large proportion of this cohort (42–46%) was taking immunomodulatory medications. This is fairly similar to frequencies reported in other MS cohorts. The associations of immunomodulatory medication use with depression state will be described in another paper. However, our evidence indicates that controlling for disability and fatigue is adequate to account for clinical variability and its association with depression in this sample.

Factors which might have impacted upon depression risk, and which would have been useful to account for, but which we did not have information, include addiction and drug use, membership in community and other organizations, and local environmental characteristics, particularly air/water and noise pollution. Addiction and drug use are obviously quite relevant, but we only queried tobacco and alcohol use. While it is possible for some covariance of illicit drug use with tobacco/alcohol, our failure to measure these exposures is a limitation. Membership in community and other social organizations could also impact upon depression, so data on this would have been a useful analysis, but one which we unfortunately cannot examine. Likewise, environmental and noise pollution would be of interest, both for overall quality of life and potentially for its impacts on physical activity and time outdoors. Future studies would be strengthened by measuring these parameters.

Another element of interest is socioeconomic status, since this can impact upon depression and modify the relationship of other factors with depression. However, we only measured this factor at follow-up, precluding a more definitive assessment of its prospective relationship, especially with change in depression state. Accordingly, we do not control for it here, though it is examined in another manuscript.

## Conclusion

In a large prospective cohort study of people with MS and depression, we have found evidence that a variety of lifestyle factors are inversely associated with depression, though of these, only alcohol, diet and supplement use were independently associated with change in depression. These results, if confirmed, suggest that some healthy lifestyle behaviors may positively impact depression risk among people living with MS.

## Ethics statement

The Health Sciences Human Ethics Sub-Committee at the University of Melbourne provided ethical approval for the study (Ethics ID: 1545102). Participants were asked to read the participant information and to consent before entering the survey.

## Availability of data and material

Data may not be shared due to the conditions approved by our institutional ethics committee, in that all data are stored as re-identifiable information at the University of Melbourne in the form of password-protected computer databases, and only the listed investigators have access to the data. All data have been reported on a group basis, summarizing the group findings rather than individual findings so personal information cannot be identified. Therefore, we can supply aggregate group data on request. Readers may contact George Jelinek or Tracey Weiland.

## Author contributions

GJ, TW, KT, SS, CM, CB, and ADL are responsible for study concept; KT drafted and edited the manuscript; SS, CM, CB, and EO contributed to cohort management and cleaned and prepared the data for analysis. SS undertook data analyses. TW, GJ, SS, SN, ADL, CB, EK, and CM contributed to editing an earlier version of the manuscript. All authors approved the final version of the manuscript.

### Conflict of interest statement

GJ receives royalties for his books, Overcoming Multiple Sclerosis and Recovering from Multiple Sclerosis. GJ, SN, and KT have received remuneration for conducting lifestyle educational workshops for people with MS. The remaining authors declare that the research was conducted in the absence of any commercial or financial relationships that could be construed as a potential conflict of interest.
